# Flipped classroom applied to Neurosurgery in undergraduate medical education

**DOI:** 10.1186/s12909-023-04158-8

**Published:** 2023-03-20

**Authors:** R Gutiérrez-González, A Zamarron, A Royuela, G Rodriguez-Boto

**Affiliations:** 1 Department of Neurosurgery, Puerta de Hierro University Hospital, IDIPHISA, Manuel de Falla 1, 28222 Majadahonda-Madrid, Spain; 2 Department of Surgery, Faculty of Medicine, Autonomous University of Madrid, Arzobispo Morcillo 4, 28029 Madrid, Spain; 3 Biostatistics Unit Puerta de Hierro University Hospital - IDIPHISA, CIBERESP, Madrid. Manuel de Falla 1, 28222 Majadahonda-Madrid, Spain

**Keywords:** Active learning, *Flipped classroom*, Formative assessment, Gamification, Problem-based learning

## Abstract

**Purpose:**

To compare the academic achievement obtained in *Neurosurgery* in a class of undergraduate students according to the pedagogical methodology employed: flipped classroom (FC) versus traditional lecture. Students’ satisfaction with the FC model is also analyzed.

**Methods:**

A quasi-experimental study was designed. The traditional lecture was the pedagogical method employed in teaching units (TUs) 1, 2, and 3 (61, 60, and 66 enrolled students, respectively), whereas TU 4 (69 enrolled students) used the FC methodology.

**Results:**

The dropout rate was lower, whereas the academic achievement and the rate of correct answers were higher in TU 4 compared to the rest of the TUs, but these results were not statistically significant. However, the mean score obtained in *Neurosurgery* was significantly higher in TU 4 compared to the rest of the TUs (*p* = 0.042). Active learning activities based on clinical cases were positively emphasized. The main weakness was with the time consumed for video-recorded lecture viewing.

**Conclusions:**

The FC approach showed better academic results than traditional lectures when comparing students in the same Medical School during the same academic year undergoing the same exam. The students rated the FC approach positively, considering it stimulating and useful for learning.

## Background

Current educational trends encourage the shift toward the learning paradigm to the detriment of the teaching paradigm. Learning is a student-centered approach, encouraging self-regulated learning and self-training abilities. The methodology is based on questions and challenges that interest and motivate the students to ensure their engagement [1]. Teachers guide in this process, and the problems are the focus and the stimulus for learning.

Traditional lecture (teacher-centered approach, where the teacher determines what is learned, how it is learned, and when and at what pace it is learned) shows the student's eminently passive and unmotivated participation. Thus, the student is reduced to the role of listener, who must assimilate and often memorize the information provided by the teacher-textbook binomial based on the paradigm of the contents [1]. In contrast, the “flipped classroom” (FC) proposes pre-class activities for direct instruction. It uses face-to-face hours for active learning methodologies as an exponent of a student-centered approach [2, 3]. Such in-class activities represent an opportunity for the students to interact with the instructor, increasing the focus on higher-order thinking skills and receiving immediate feedback, which may result in higher student motivation and engagement [4].

The concept of FC was consolidated by Bergman and Sams [5], who planned a new methodology to teach chemistry lessons that could reach all students. They noticed low assistance and a high rate of missing classes in a relatively rural school. In 2007 they designed PowerPoint recordings converted into videos and distributed them online to reach all students. They realized that the videos were used by those students who had missed the classes but by most of the students. Thus, they progressively changed their methodology and submitted the videos before the classes as a homework activity, keeping the class time for helping students with the concepts they had yet to understand.

Since that moment, FC has been applied to different educational levels and academic domains [6]. Although little formal data is available, several studies have shown better achievement in different scenarios [4, 7]. Thus, Fulton et al. evidenced an increase from 29.9% to 73.8% in the percentage of students passing high school state mathematics tests after FC application. Finkel et al. evidenced a decrease from 44 to 13% in mathematics failure rate after introducing the FC in high school. Other examples in higher education have also pointed to a deeper understanding and improved skills [4, 7]. Nevertheless, not only students’ experience has been analyzed. A survey compiling the experience of 450 teachers that applied FC showed that 67% referred to improvement in test scores, 80% informed about better students’ attitudes towards learning, and 99% confirmed they would use the methodology in the following course [4].

There has been a rising trend in research in the last decade, measured by the number of published studies [8]. That interest in the effectiveness of FC affects almost all health profession specialties [8, 9]. Medical education seems perfect for this pedagogical methodology since students are usually assumed to be highly motivated and “life-long learners.” Thus, several studies have focused on undergraduate medical students and postgraduate training doctors, too [10–14]. There is considerable consensus on the preference of medical students for the FC approach since it increases motivation and engagement [8, 10, 15]. However, the evidence of the actual impact on learning is less clear. Reliable tools must be designed to measure such influence, and long-term effects must be considered [8, 10, 12, 14, 15].

Only two studies have been published to date regarding the effect of FC in Neurosurgery [16, 17]. One was performed with undergraduate students and suggested that FC methodology is preferred. However, it only included two topics of the whole subject and focused on students’ satisfaction, missing the assessment of academic achievement [16]. Another experience with postgraduate residents, based on case discussions, showed better achievement when compared with the traditional method used the previous years [17]. Notably, the cognitive level of graduate doctors is higher than undergraduate students, preventing extrapolating conclusions.

Recent meta-analysis and systematic reviews suggest that the FC methodology, based on active learning activities, improves student learning, performance, skills, and satisfaction in health professions education [8, 9, 18, 19]. However, more solid evidence of its effect on knowledge retention and transfer to clinical practice is needed [10].

This study aims to compare the academic achievement obtained in *Neurosurgery* in a class of undergraduate students according to the pedagogical methodology employed: FC versus traditional lecture. The ultimate purpose is to demonstrate if the FC is superior to the traditional lecture model in undergraduate neurosurgical students. In addition, it aims to analyze the satisfaction of the students who studied under the FC model. Unlike most of the studies published to date on this subject, this work adds information on student participation in the different activities designed as part of the FC methodology. It may allow us to follow up on the degree of engagement in future academic years and compare data with other studies.

## Methods

### Study design and subjects

A quasi-experimental study was designed. The project was accomplished in the 2021/2022 academic year and only affected the subject of *Neurosurgery*, which forms part, together with *Neurology*, of the compulsory topic “Medicine and Surgery of the Nervous System.” This subject is taught to fourth-year undergraduate medical students. The study was approved by the local Ethics Committee and was conducted following the 1964 Helsinki Declaration and its later amendments or comparable ethical standards.

The School of Medicine comprises four teaching units (TUs), each assigned to a different hospital. Students choose the TU before starting the first course according to the cut-off score obtained, and they keep that assignment during the six courses that medical education lasts in our country.

The content of the subject is the same for all the TUs. Likewise, the exam is the same for all students and is accomplished simultaneously in a single exam session. The subject Coordinator is responsible for the design of the multiple-choice exam. Even though the contents are similar for all students, each TU is independent (except for the final exam), so each teacher chooses the tools and methodology applied during the course. The traditional lecture was the pedagogical method employed in TUs 1, 2, and 3 (61, 60, and 66 enrolled students, respectively), whereas TU 4 (69 enrolled students) used for the first time the FC methodology for the complete curriculum of *Neurosurgery*.

A single teacher with the category of Associate Professor of Health Sciences (first author) was responsible for flipping the *Neurosurgery* classroom on TU 4. It was her first experience with this pedagogical methodology. All feedback was provided by the same teacher, thus unifying criteria. In the rest of the TUs, traditional lectures were taught by between 2 and 4 professors, depending on the corresponding TU, all with the category of Associated Professor of Health Sciences except for the subject Coordinator (Full Professor).

The study’s primary outcome included the impact on learning (measured as academic results), whereas the secondary outcome was students’ satisfaction with FC pedagogical methodology.

### Traditional lecture

The same methodology was used in all classroom sessions (4 h of seminars and 7 h of theoretical classes), based on traditional lectures taught by accredited teachers and supported by a PowerPoint presentation distributed to the students at the end of each session. Seminars required compulsory attendance, while it was free in the rest of the classes.

### Flipped classroom

At the beginning of the course, students received an e-mail through the virtual campus explaining and organizing how the learning would take place and what the teaching–learning processes would be both inside and outside the classroom. Evaluation criteria for the activities used throughout the course were also provided (rubrics and evaluation scales). Finally, indications on how to set up the working groups during the seminars were given too.

Before each face-to-face class, the teacher provided the students with the theoretical content they would later work on in the classroom through the *Edpuzzle* platform. It allows the teacher to create a class and assign homework to the students, using video lessons. The tool also allows inserting questions and notes throughout the clip to provide immediate feedback after answering them. After the assignment, the teacher can access to data about students’ participation. Thus, the students had online access to the video-recorded lectures for 15 days for each video. Every class was synthesized in a variable number of videos (from 3 to 7), most of them of short duration (3–7 min), and with short quizzes inserted throughout the clip (between 1 and 3). Eventually, review scientific papers were also electronically sent to all students. Thus, each face-to-face class began briefly explaining the learning objectives and their interrelation with previous topics of the same or other subjects. A problem-based learning activity followed this reflection in the hours assigned to seminars (4 h; mandatory attendance), and a puzzle-type activity in the hours allocated to theoretical classes (7 h; attendance recommended but not required).

#### Seminars

One week in advance, each working group received a clinical case representative of the topic to be covered in each class as a starting point to work on the acquisition of specific knowledge but also to encourage cooperative work and critical thinking. The students had to answer the 5–7 questions posed. Each group had to meet to work on the solution to the problem and share the result with all the students during the classroom session. The choice of the student who presented the clinical case and answered the questions was random. Each group also had to submit a word or pdf document compiling all the answers to the questions posed. The structure was very similar in all clinical cases (symptoms and neurological exam, diagnostic exams, differential diagnosis, treatment, outcome) but with specific differential features. A rubric provided at the beginning of the course was used as a consultation tool. The submission was mandatory to access the final evaluation, and although it was not scored, the teacher corrected it, providing collective feedback.

#### Theoretical classes

After the teacher reflected on the learning objectives, a puzzle (active and cooperative learning activity) was developed by distributing three different clinical cases that allowed the development of the advanced content in the video-recorded lectures. After individual reading of the material by each student, all the students with the same clinical case met for discussion (expert teams) and resolution of the 5–7 questions posed. Finally, each clinical case was read and discussed in common with the whole class for its correction, providing immediate feedback.

Each face-to-face session would end with a gamification activity designed with the *Wooclap* tool that would review the main concepts of each class. *Wooclap* is an interactive electronic platform used to create polls and questionnaires that can be solved self-paced or in real-time. The site's user answers questions anonymously through a technology device. The primary purpose, in this case, was to provide feedback about the concepts learned during the class. However, due to a lack of time at the end of each class, this activity was redesigned and adapted, so the teacher provided a link by e-mail to complete the questionnaires (multiple-choice test) as self-paced after-class work. An example of a multiple-choice question asked for the topic on brain tumors is: “A tumor located in the pineal region may cause the following syndrome: a-Weber syndrome; b-Millard-Gubler syndrome; c-Parinaud syndrome; d-Wallenberg syndrome.” Similarly, an example of an open-ended question about spinal tumors is: “What drug is recommended to reduce pain in osteoid osteoma?”.

At the end of the last seminar, the students completed an anonymous Likert-scale-based survey to evaluate the teaching and learning process and the pedagogical model used. The questionnaire consisted of 13 questions to be rated according to a 5-point scale, where 1 = "strongly disagree with the statement" and 5 = "strongly agree with the statement." In addition, the survey included two free-response questions to detect the strengths and weaknesses of the model.

### Assessment of the impact on learning

The academic results evaluated were the *dropout rate* (number of students not taking the exam compared to the total number of students enrolled in the subject); the *pass rate* (number of students passing the multi-choice exam compared to the total number of students taking the exam); the *academic achievement* (number of credits passed out of the total number of enrolled credits); the *correct answer rate* (number of correct answers out of the total number of specific Neurosurgery questions -26-); and the *average score* of the Neurosurgery questions out of 26 possible points. For this calculation, correctly answered questions added 1 point, unanswered questions added 0 points, and failed questions subtracted 0.33 points.

Students’ satisfaction with the FC model was evaluated with the Likert-scale-based survey abovementioned.

### Statistical analysis

Database information was processed and analyzed using StataCorp. 2019 (*Stata Statistical Software: Release 16*. College Station, TX: StataCorp LLC). Numerical variables represented by the mean and standard deviation (SD) were contrasted with the Student-T test or Mann–Whitney U test if the normality assumption was not satisfied between the two comparison groups. The Chi-square test was used to contrast categorical variables and absolute and relative frequencies as the description measure. The considered level of significance was 5%. All *p* values were based on two-tailed tests of significance.

## Results

A total of 256 students were enrolled in the study across TUs 1–4, of which 69 participated in the TU 4 FC methodology (26.95%). The remaining 73.05% attended traditional lectures. Female students entailed 73.83% of the sample. The proportion male:female was not significantly different among groups (*p* = 0.534).

Concerning the FC, the course planning was adequate, and the deadlines were met when generating the materials, sharing them, and providing feedback to the students after different activities.

### Video-recorded lectures viewing

**Fig. 1:**
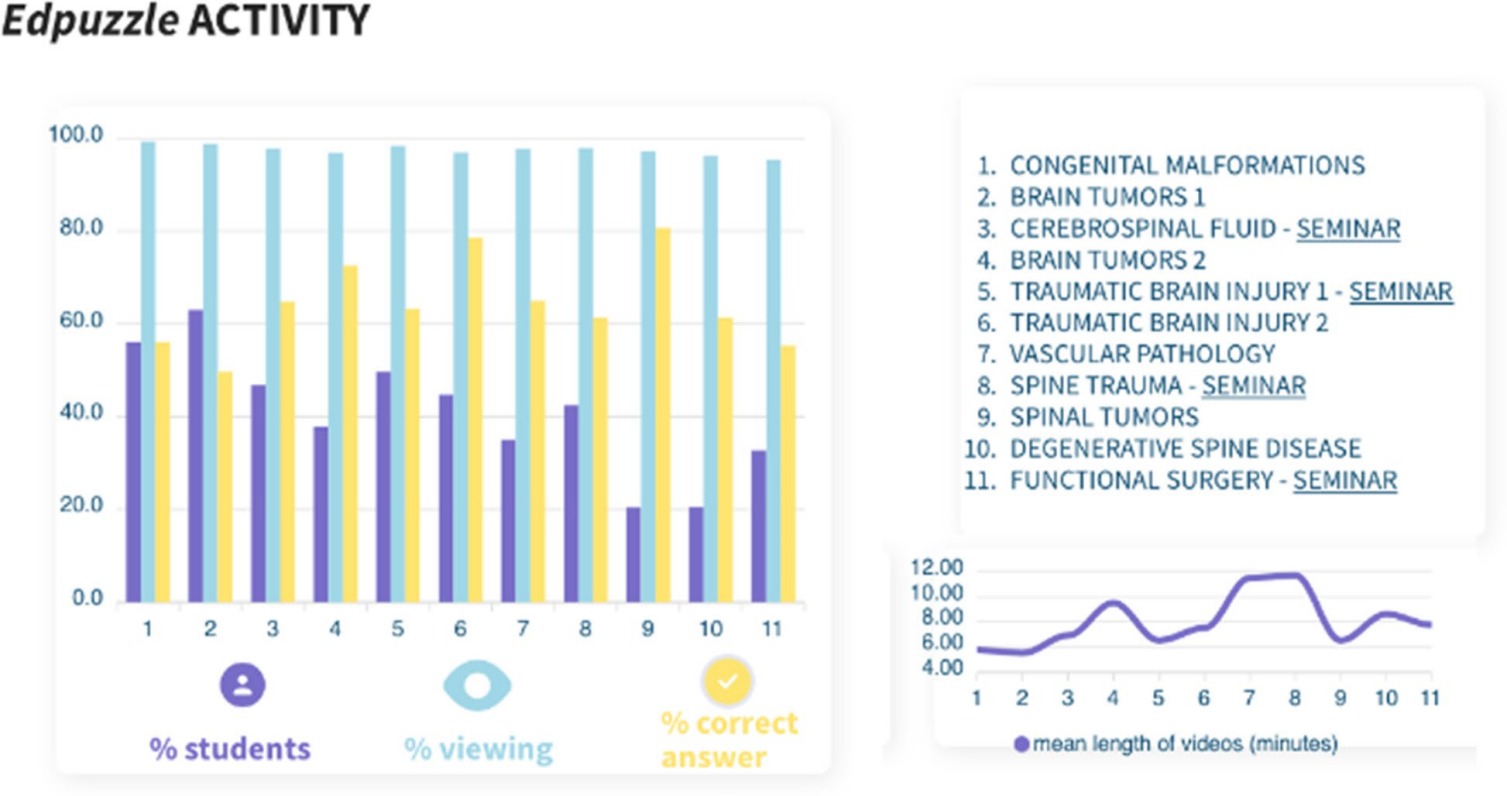
Summary of students’ participation in the *Edpuzzle* activity. The table (left) shows the percentage of all students who signed up for the platform (55) that viewed at least one video of each topic (purple); the percentage of all videos that they viewed at least for one second of each topic (blue); the percentage of questions included in the videos correctly answered (yellow). A table of contents is added (right) with each theme’s respective mean length of videos (below). Thus, 20% of all students that signed up for *Edpuzzle* (55) viewed at least one video of topic number 9, “spinal tumors.” They viewed at least one minute of virtually all videos on that topic, and correctly answered 80% of all questions included in the videos of that specific theme

Fifty-one tasks corresponding to the videos of the 11 classes were included. Fifty-five students out of the 69 enrolled in the course registered on the *Edpuzzle* platform for video viewing. Fifteen percent of the registered students did not perform any connection or viewing, so 68% of all the students (47 out of 69) actively participated in the event. The average time spent to complete the tasks by the remaining 85% was 4 h and 26 min. The overall correct answer rate for the questions inserted in the videos was 63.7%. A correct answer rate under 20% was identified in two questionnaires. Figure 1 summarizes the participation in the activities generated with the *Edpuzzle* tool.

### Submission of seminar activity

All groups submitted the four seminars’ clinical case solutions on time. Students received collective feedback via e-mail from the teacher.

### Gamification

**Fig. 2:**
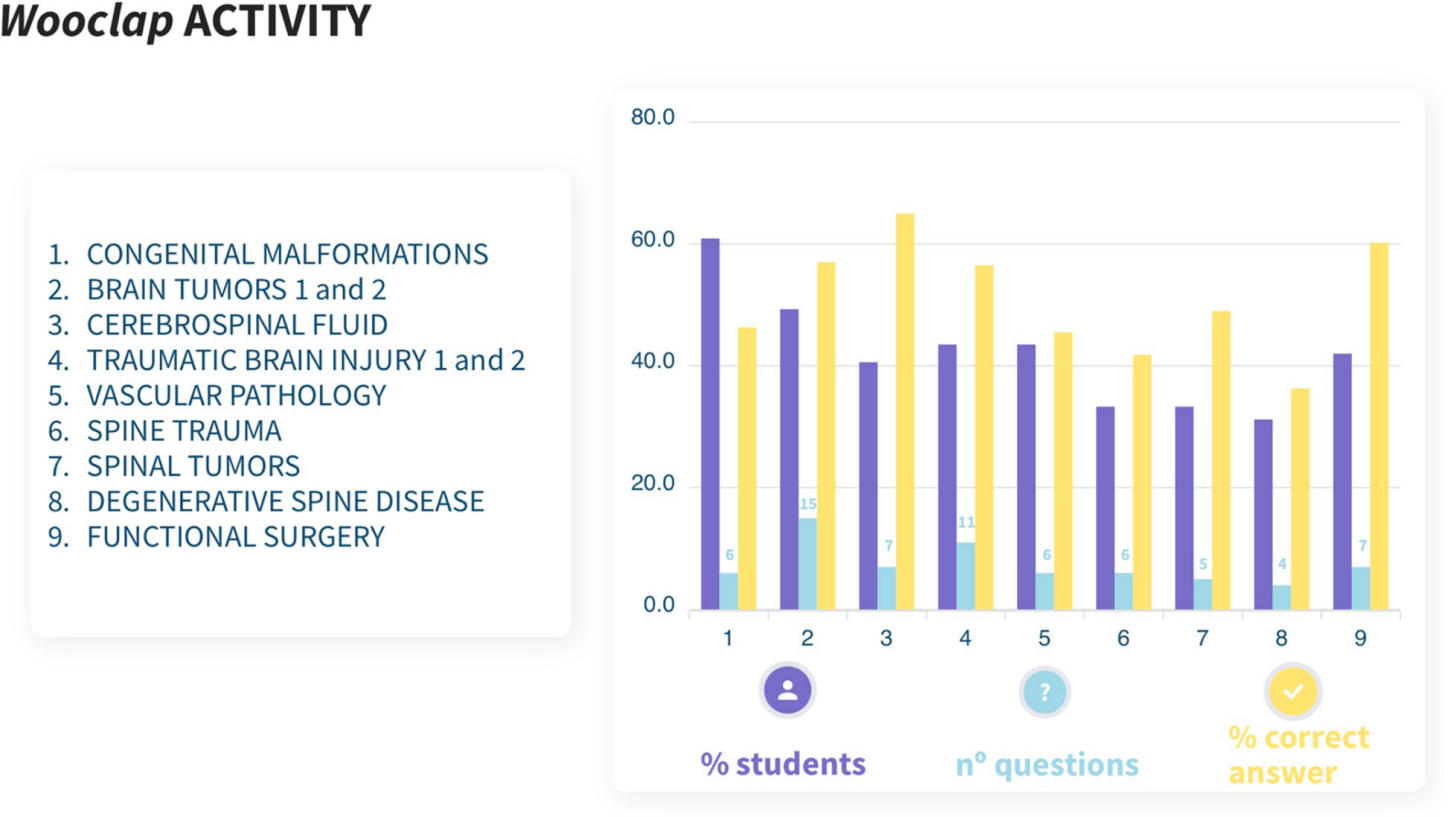
Summary of students’ participation in the *Wooclap* activity. The table (right) shows the percentage of all students enrolled in the course (69) that completed the questionnaire of each topic (purple); the number of questions of each questionnaire (blue); the percentage of questions correctly answered in each theme (yellow). A table of contents is added (left)

The *Wooclap* tool was used to generate nine questionnaires (brain tumors and traumatic brain injury required two classes each, but only one questionnaire for topic) with an average of 7.4 questions each. An average of 29 students participated in each questionnaire (42.02% of the total students enrolled). The overall correct answer rate was 52.7%, but a decrease under 20% was identified in three questions. Figure 2 summarizes the participation in the *Wooclap* activity.

**Fig. 3:**
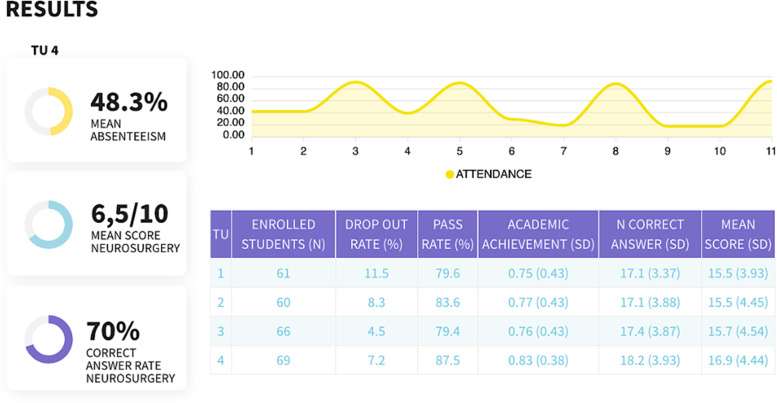
Summary of academic results. The table (inferior) shows the number of enrolled students, dropout rate, exam pass rate, academic performance, number of correct answers, and mean score in the final exam (the two latter, only related to the *Neurosurgery* curriculum) corresponding to each of the four teaching units (TUs). Students' attendance at each face-to-face session is also registered as a percentage of all enrolled students (superior graph)

The academic results obtained by the students are summarized in Fig. 3 and Table 1 and revealed a dropout rate of 7.25% in TU 4 compared to 8.02% in the rest of the TUs. Of the 64 students taking the exam in TU 4, 87.50% passed the multi-choice exam of the subject "Medicine and Surgery of the Nervous System." This rate was higher than in the rest of the TUs. The academic achievement in the subject was 0.83 in TU 4, also higher than the 0.76 achieved in the rest of the TUs. However, none of these results accomplished statistical significance. The rate of correct answers in the specific part of *Neurosurgery* reached 70% in TU 4 compared to 66.15% in the rest of the TUs (*p* = 0.079). Finally, the mean score obtained in the specific area of *Neurosurgery* was significantly higher in TU 4 (16.88/26) compared to the rest of the TUs (15.59/26).

**Table 1:** Comparison of outcome variables

Variable	FLIPPED CLASSROOM (*n* = 69)	TRADITIONAL LECTURE (*n* = 187)	STATISTICAL TOOL (VALUE)	*p* value
Drop-out rate	5 (7.25)	15 (8.02)	X^2^ (Pearson-chi2 = 0.0420)	*p* = 0.838
Pass rate n, (%)	56 (87.50)	139 (80.81)	X^2^ (Pearson-chi2 = 1.4526)	*p* = 0.228
Mean academic achievement (SD)	0.826 (0.381)	0.759 (0.429)	U Mann–Whitney (z = -1.136)	*p* = 0.256
Mean correct answers (Neurosurgery) (SD)	18.203 (3.933)	17.232 (3.706)	Student-T (t = -1.7590)	*p* = 0.079
Mean score Neurosurgery (SD)	16.883 (4.438)	15.589 (4.303)	Student-T (t = -2.0370)	*p* = 0.043

**Fig. 4:**
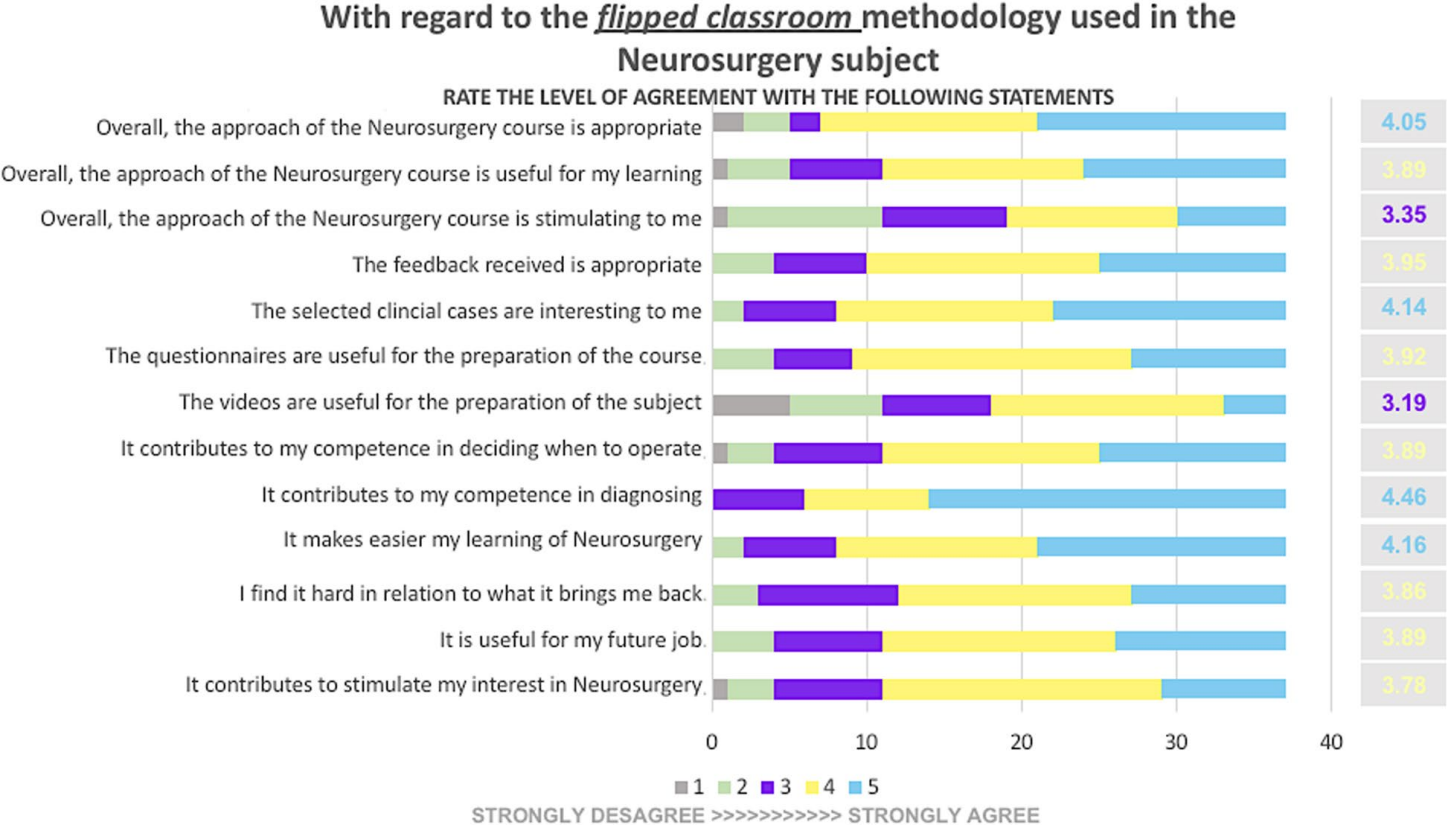
Summary of the satisfaction survey students from TU 4 filled out at the end of the course. Statements are shown on the left, and average scores appear on the right. In the center, the number of students who assigned each statement a given rank

To assess the level of satisfaction with the FC methodology of students from TU 4, 53.6% of the 69 enrolled students answered the questionnaire distributed online at the end of the last seminar. Thus, the students rated the FC approach positively, considering it stimulating and useful for learning (Fig. 4).

The active learning activities based on clinical cases (problem-based learning) and the involvement of the teacher were positively emphasized. Only one student highlighted the usefulness of the questions inserted in the videos in the open questions of the survey.

**Fig. 5:**
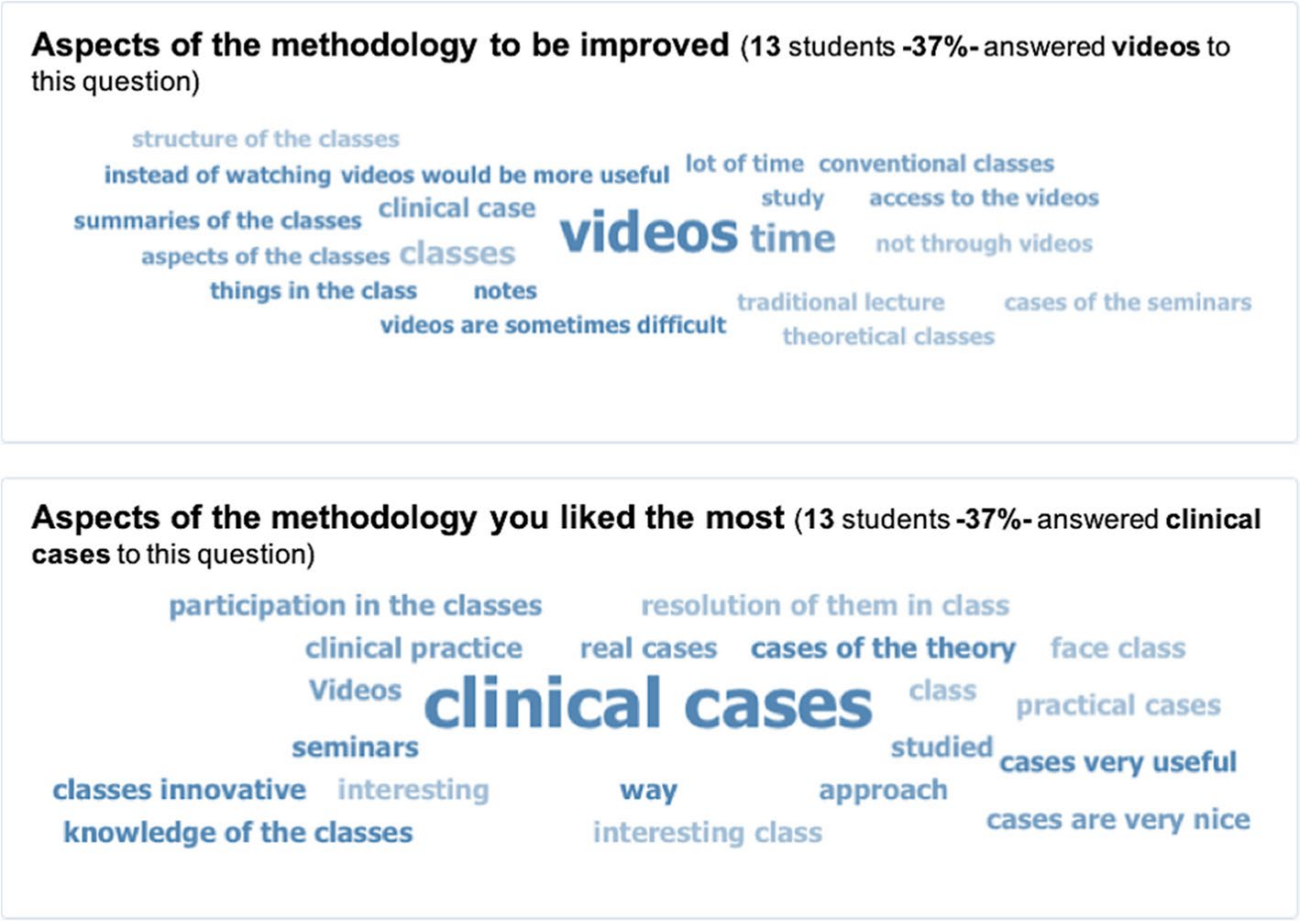
Word cloud for the two free-response questions included in the survey

The main area for improvement was the video lessons, according to 37% of the students that answered the survey (Fig. 5). They mentioned difficulties such as the time consumed for video-recorded lecture viewing (19%) or the need to watch the entire video again each time it was rewound to take notes (8%).

## Discussion

This project intends a radical change in the teaching methodology used so far by our team, whose pillar was the traditional lecture. All the methods and tools included in this study focus on the active role of the students in their learning process. Thus, they are oriented toward students learning to solve the clinical cases they will face, regardless of the specialty they choose in the future. But not only that. The methodologies also point towards competency-based training. However, it is difficult to find studies that evaluate results by competencies such as critical or creative thinking due to the difficulty of designing objective tools to measure these skills.

The literature supports the benefit of active learning [1, 2, 16]. The FC is based on different pre-class, in-class, and after-class activities, properly integrated and aligned with clear learning objectives that the students must understand and recognize [20]. The most common preferred pre-class activities include textbooks, instructor-developed reading material, or videos [20]. Narrated PowerPoint videos were the material we most used at this stage since we considered it would replace traditional lectures (students sometimes reject profound changes), but it would add the possibility of consulting them as much as needed. The script of the slides allowed for a cleaner design, without so much text and with more schemes and concept maps. The participation in this activity was moderate but satisfactory, considering the availability of other years’ and other schools’ notes on the net. One of the most critical factors that affected students’ satisfaction was the quality and time-consuming of pre-class videos, as well as the impossibility, as the activity was designed, to fast forward or rewinding the video whenever necessary, forcing it to be watched entirely. However, the insertion of multi-choice questions to engage the pupil and provide quick feedback was appreciated, as observed in other studies [8, 16]. Additional scientific articles were delivered not only to fill some topics but to make students get in touch with a tool they will use as future doctors. No data were recorded on the number of consultations in these texts so no conclusion can be drawn. However, since *Neurosurgery* only involves one-third of the whole subject, this material can workload students more than help them at this point.

In-class work frequently uses problem-solving activities, moreover in medical education. Problem-based learning has sufficient theoretical and practical support to become an effective alternative to the paradigm of the contents for developing key competencies [21]. It is the starting point of a stimulating educational experience (since it requires a certain cognitive effort to answer the questions of the problem) that involves collaboration with other students to solve complex issues through debate and argumentation. In our experience, it also allowed the teacher to introduce concept maps, diagrams, and management schemes to support and integrate the information. Clinical cases are perfect examples of meaningful learning since medical practice is mostly based on solving "problems" for our patients [21]. Be considered that this is our students' first experience with FC and that the subject is taught in their fourth year (the first three years they study basic sciences) when they have their first contact with patients during practical workshops. We agree with other authors that this approach increases student motivation, interest, and engagement [1, 16]. Real examples highlight the relevance of the topics and the connection between pre-class and in-class activities [20].

The teamwork design handles two aspects. On the one hand, the total number of students reached 69 with mandatory attendance (seminars). On the other hand, even though a doctor can solve a clinical case alone, the usual practice is teamwork and discussing cases in uni- or interdisciplinary clinical sessions. When students learn to work in a team and consult with colleagues, they train valuable skills for their professional future (competency-based training). Creating learning networks is one of the main benefits of collaborative, cooperative learning, which makes such learning stronger and more lasting and, therefore, more meaningful. The aim is to achieve the best result for the student and other classmates, thus establishing a positive interdependence. It allows the development of the student's values, abilities, and skills and improves academic results and motivation [1, 22, 23]. Attendance was limited when it was optional despite students’ acceptance of the clinical cases approach and cooperative learning, according to the satisfaction survey. Other subjects taught synchronically also showed low attendance, which may be related to the end of the academic year and the proximity of final exams.

Other in-class activities are projects, quizzes, or guided questions. The benefits of gamification in education include progress monitoring or motivation encouragement. This activity may be used in the three stages of the FC. We finally used it as after-class work, avoiding competition with other classmates, despite initial planning. We find it helpful to encourage self-esteem and self-criticism in a safe environment for students without penalties for error [24]. Besides that, it is a great instrument to provide feedback and detect deficiencies or challenging concepts. Both platforms used in this project (*Edpuzzle* and *Wooclap*) allowed us to find those questionnaires with a low success rate, which may lead to reformulating a question or better explaining a topic in the future. Finally, after-class work also allows the spaced evocation of contents, a key fact in learning [20].

Feedback may be the principal tool in the learning paradigm [25]. Thus, it should be timely (avoid delay), constructive (respectful of the student's self-esteem), and should focus on the needs of the student (the person who is learning) [26]. The FC may facilitate students the opportunity, the space, and the moment to receive effective feedback [6]. We paid special attention to it, including feedback during different moments of the learning process (video-inserted questions, problem-based learning, gamification). The FC made possible a very close relationship with those students that demanded it, having easy access to the instructor. Students also appreciated it, according to the satisfaction survey. Similarly, Bergman and Sams [5] noted that teachers who circulate the classroom and talk to students are likely to understand better and respond to their students' emotional and learning needs.

The assessment of what the student has learned is also an important issue. Most studies employ test scores to evaluate academic achievement, but tests may need to be more accurate with skills or competencies acquisition [20]. Specific tools must be designed to assess other aspects beyond content. However, this is time-consuming and prevents comparison with traditional methodologies or previous experience.

The academic results obtained in this study support the use of the FC approach. All outcomes were better in the FC cohort than in the traditional lecture. Considering specific questions of *Neurosurgery*, the final mean score was significantly higher with the FC methodology compared to traditional lectures. Other studies in undergraduate medical education have shown similar results when teaching basic or clinical subjects such as physiology, pathophysiology, or hematology [27–29]. However, most researchers apply the FC methodology only to specific topics of a determined subject [28–30]. Thus, a relevant contribution of this research from a theoretical point of view is the validation of the methodology for the whole curriculum of *Neurosurgery* in undergraduate students, in contrast to other studies focused on specific issues or performed with postgraduate residents [16, 17]. From a practical point of view, it demonstrates that it is an acceptable methodology, which is applicable regardless of the teacher's experience, that achieves academic results at least equal to the traditional lecture.

## Conclusions

This is the first study performed on undergraduate students that shows that flipping the whole curriculum of *Neurosurgery* and obtaining good academic achievement is feasible. It also adds valuable information on student participation in the different activities included. The results now obtained must be confirmed in further academic years.

The limitations of this research include the lack of randomization, even though the selection of the TU to which each student was assigned occurred a priori (without teacher’s intervention), as abovementioned. Other issues to be considered involve possible confounding factors (cohort homogeneity regarding the number of repeaters or instructors’ years of experience) and sample size. The specific interest of students in the subject is unknown and may also interfere with participation and motivation. Future directions point to validating the observed differences, improving the material's quality, and designing specific tools to assess knowledge, skills, and competencies.

## Data Availability

The datasets used and/or analyzed during the current study are available from the corresponding author on reasonable request.
